# Medición de la concentración de ertapenem en el plasma y líquido ascítico mediante UHPLC-MS/MS. Aplicación en pacientes cirróticos con peritonitis bacteriana espontánea

**DOI:** 10.1515/almed-2023-0122

**Published:** 2023-11-21

**Authors:** Raúl Rigo-Bonnin, Alberto Amador, María Núñez-Gárate, Virgínia Mas-Bosch, Ariadna Padullés, Sara Cobo-Sacristán, José Castellote

**Affiliations:** Laboratorio Clínico, Hospital Universitario de Bellvitge, Instituto de Investigación Biomédica de Bellvitge (IDIBELL), Hospitalet de Llobregat, Barcelona, España; Servicio de Farmacia, Hospital Universitario de Bellvitge, Instituto de Investigación Biomédica de Bellvitge (IDIBELL), Hospitalet de Llobregat, Barcelona, España; Unidad de Hepatología y Trasplante Hepático, Servicio de Aparato Digestivo, Instituto de Investigación Biomédica de Bellvitge (IDIBELL), Hospitalet de Llobregat, Barcelona, España

**Keywords:** ertapenem, líquido ascítico, plasma, cromatografía líquida de alta y rápida eficacia acoplada a la espectrometría de masas en tándem (UHPLC-MS/MS)

## Abstract

**Objetivos:**

La peritonitis bacteriana espontánea es una complicación frecuente y grave de los pacientes cirróticos con ascitis. Actualmente, los antibióticos carbapenémicos son el tratamiento de elección en pacientes con peritonitis nosocomiales o relacionadas con el sistema sanitario. Pese a ello, los estudios de eficacia del ertapenem en pacientes cirróticos con peritonitis bacteriana espontánea son limitados y la farmacocinética y farmacodinamia de este antibiótico continúa siendo desconocida. Así, el objetivo de este estudio es desarrollar y validar procedimientos de medida basados en la cromatografía líquida de alta y rápida eficacia acoplada a la espectrometría de masas en tándem (UHPLC-MS/MS) para medir las concentraciones de ertapenem en el plasma y en el líquido ascítico.

**Métodos:**

El pretratamiento de las muestras se realiza utilizando una precipitación de proteínas con acetonitrilo. La separación cromatográfica se lleva a cabo en una columna C_18_ de fase inversa Acquity^®^-UPLC^®^-BEH^TM^ (2,1 × 100 mm id, 1,7 µm) utilizando un gradiente no lineal de agua/acetonitrilo que contiene un 0,1 % de ácido fórmico y an un flujo de 0,4 mL/min. El ertapenem y su patrón interno (ertapenem-D_4_) son detectados mediante espectrometría de masas en tándem en las modalidades de ionización mediante electroespray positiva y de monitorización múltiple de reacción utilizando, como transiciones de masa, 476,2→346,0/432,2 para el ertapenem y 480,2→350,0 para su patrón interno.

**Resultados:**

No se observan interferencias ni contaminación por arrastre significativas. Las imprecisiones, los sesgos relativos absolutos, así como los efectos matriz y recuperaciones normalizadas son ≤14,5 %, ≤9,3 %, (92,8−104,5) % y (98,8−105,8) %, respectivamente. Los procedimientos de medida cromatográficos son lineales entre (0,50−100) mg/L.

**Conclusiones:**

Los procedimientos de medida basados en la UHPLC-MS/MS desarrollados y validados podrían ser de utilidad para realizar estudios farmacocinéticos y farmacodinámicos en sujetos con cirrosis hepática que presentan peritonitis bacteriana espontánea tratados con ertapenem.

## Introducción

La peritonitis bacteriana espontánea (PBE) es una complicación frecuente en pacientes con cirrosis. Se define como la infección del líquido ascítico en pacientes con cirrosis hepática en ausencia de causa secundaria. El diagnóstico se realiza mediante paracentesis con hallazgo de más de 250 células polimorfonucleares en el líquido ascítico con o sin cultivo positivo [1, 2]. Los gérmenes más frecuentemente implicados son bacterias gramnegativas, y en los últimos años ha aumentado la incidencia de infecciones por bacterias multiresistentes, factor que complica el tratamiento y aumenta la morbimortalidad de los pacientes con cirrosis hepática [3], [4], [5], [6]. Las guías actuales recomiendan, en pacientes con PBE relacionada con el sistema sanitario o nosocomial, el tratamiento con carbapenémico con o sin glicopéptido asociado, respectivamente [1, 2, 7].

El ertapenem (ETP) es un antibiótico carbapenémico aprobado para el tratamiento de infecciones intraabdominales graves, así como para las infecciones producidas por enterobacterias multirresistentes [6, 7]. A nivel farmacocinético, la actividad bactericida del ETP es tiempo-dependiente y el índice farmacocinético/farmacodinámico (PK/PD) que más se correlaciona con su eficacia es el porcentaje del tiempo durante el cual la concentración no unida (“libre”) del antibiótico no unida a proteína (“libre”) permanece por encima del valor de la concentración mínima inhibitoria o CMI (*f*T>CMI) entre dosis consecutivas. Se asume que este porcentaje debe de ser cercano al 50 %, si bien en situaciones particulares (p. ej., en infecciones por microorganismos resistentes con CMIs elevadas) se pueden requerir intervalos de tiempo mayores (70−100) [8, 9].

Actualmente, existen varios estudios relacionados con la medición de la concentración de ETP en el plasma mediante cromatografía líquida de alta (y rápida) eficacia ((U)HPLC) acoplada a la espectrometría de masas en tándem (MS/MS) [10], [11], [12], [13], [14], [15], [16], [17] pero, hasta donde sabemos, ninguno de ellos incluye la medición de la concentración de ETP en líquido ascítico en un contexto de pacientes cirróticos con PBE. Por ello, el objetivo de este estudio es desarrollar y validar procedimientos de medida basados en la cromatografía líquida de alta y rápida eficacia acoplada a la espectrometría de masas en tándem (UHPLC-MS/MS) para medir la concentración de masa de ETP en el plasma y en el líquido ascítico.

## Materiales y métodos

### Reactivos

Se utilizan los materiales de referencia certificados *Ertapenem sodium salt* (Nº cat. C3377; pureza del 98,2 %) y *[* ^*2*^ *H*_*4*_ *]-Ertapenem sodium salt* (Nº cat. C3376; pureza del 95,5 %; enriquecimiento isotópico del 98,5 %) de Alsachim (Illkirch Graffenstaden, Francia).

Se emplean los disolventes acetonitrilo, ácido fórmico, agua y metanol de calidad LC-MS/MS de Merck Millipore Group (Darmstadt, Alemania).

### Recogida de muestras de plasma y líquido ascítico que no contienen ertapenem

Se utilizan muestras de plasma y de líquido ascítico que llegan al laboratorio de urgencias de nuestro hospital. Las muestras de sangre y de líquido ascítico se recogen en tubos de plasma Vacuette^®^ de 4 mL con heparina de litio (Greiner Bio-One GmbH, Kremsmünster, Austria). Posteriormente, los tubos se centrifugan a 2000 *g* durante 10 min a temperatura ambiente. Los sobrenadantes resultantes se almacenan en tubos de polipropileno de 2 mL a (−75 ± 3) °C hasta su uso. Previamente, se separa una alícuota de cada líquido biológico para confirmar la ausencia de ETP utilizando procedimientos de medida basados en la UHPLC-MS/MS.

### Preparación de materiales de calibración, materiales de control interno de la calidad y solución de trabajo de patrón interno

Se preparan dos soluciones primarias de 1 g/L que contienen ETP a partir del material *Ertapenem sodium salt*. La primera solución se emplea para preparar los materiales de calibración y, la segunda, para los materiales de control interno de la calidad (QC). Posteriormente, se elaboran varias soluciones acuosas secundarias a partir de la primaria a valores comprendidos entre (5−1,000) mg/L y se conservan protegidas de la luz a (−75 ± 3) °C. Finalmente, cada día que se procesan muestras de pacientes, se preparan nueve materiales de calibración de ETP a valores de 0,50; 3,50; 7,50; 15,0; 25,0; 40,0; 60,0; 80,0 y 100 mg/L diluyendo, en una proporción de 1:9, las correspondientes soluciones secundarias con muestras de plasma o líquido ascítico que no contienen ETP.

Los QC se preparan y conservan de forma similar a los materiales de calibración, utilizando la segunda solución primaria de ETP. Los QC de plasma y líquido ascítico se elaboran a valores de 1,50; 10,0; 50,0 y 75,0 mg/L.

La solución primaria de patrón interno (Ertapenem-D_4_) se realiza a 1 mg/L en metanol a partir del material *[* ^*2*^ *H*_*4*_ *]-Ertapenem sodium salt*. Esta solución se almacena protegida de la luz a (−75 ± 3) °C. La solución de trabajo de patrón interno (PI) se prepara añadiendo 300 µL de la solución primaria en 10 mL de acetonitrilo (30 mg/L).

### Instrumentación

Los análisis se llevan a cabo con un sistema cromatográfico Acquity^®^-UPLC^®^ acoplado an un espectrómetro de masas de triple cuadrupolo Acquity^®^-TQD^®^ (Waters, Milford, MA, EEUU).

### Condiciones cromatográficas

La separación cromatográfica se realiza utilizando una columna de fase inversa Acquity^®^-UPLC^®^ BEH™ C_18_ (2,1 × 100 mm id, 1,7 µm) de Waters, y manteniendo el compartimiento de la columna a 40 °C.

Se aplica una elución en gradiente de la fase móvil formada de un disolvente A (fase móvil A), compuesto de 0,1 % (*v/v*) de ácido fórmico en agua, y un disolvente B (fase móvil B) que contiene 0,1 % (v/v) de ácido fórmico en acetonitrilo (Tabla 1). La temperatura del muestreador automático se mantiene a (15 ± 1) °C.

**Tabla 1:** Elución cromatográfica.

Paso	Tiempo total, min	Fase móvil A, %	Fase móvil B, %	Flujo, mL/min	Tipo de elución
1	0,0	98	2	0,4	Isocrática
2	0,4	50	50	0,4	Gradiente lineal
3	2,0	98	2	0,4	Gradiente no lineal

Fase móvil A: ácido fórmico 0,1 % (*v/v*) en agua. Fase móvil B: ácido fórmico 0,1 % (*v/v*) en acetonitrilo.

### Condiciones del espectrómetro de masas

Los parámetros genéricos del espectrómetro de masas para el ETP y su PI son: potencial de capilaridad, 1,5 kV; potencial de la fuente extractora, 3 V; potencial de la lente de radiofrecuencia, 0,1 V; temperatura de la fuente de ionización, 125 °C; temperatura de desolvatación, 450 °C; flujo de gas de desolvatación, 800 L/h; flujo de gas del cono, 50 L/h; y el flujo del gas de colisión, 0,20 mL/min. Como gas de nebulización y de desolvatación se emplea nitrógeno, y argón como gas de colisión.

La detección de ETP y de su IS (ETP-D_4_) se lleva a cabo trabajando en las modalidades de monitorización múltiple de reacción (MRM) y de ionización mediante electrospray positiva (ESI+) verificando la formación del aducto [ETP-H]^+^. Para el ETP, se utiliza una transición de masas de 476,2→346,0 para la cuantificación y de 476,2→432,2 para la comprobación (cualificación). Para el ETP-D_4_, sólo se emplea la transición 480,2→350,0. El potencial de cono aplicado es de 25 V para el ETP y su PI, y las energías de colisión son de 15 eV para la primera transición de ETP y su PI, y de 25 eV para la segunda. El tiempo de permanencia o “dwell time” para cada compuesto se selecciona en 100 ms.

### Pretratamiento de las muestras

Se transfirieren 100 µL de materiales de calibración, QC, muestras de plasma o de líquido ascítico en tubos de microcentrífuga de polipropileno de 1,5 mL. Después, se realiza una precipitación de proteínas añadiendo 300 μL de la solución de trabajo de PI, seguida de una homogenización en un vórtex durante 2 min, y una centrifugación posterior durante 5 min a 11,000 *g* a temperatura ambiente. Finalmente, se diluyen 100 μL del sobrenadante resultante con 400 μL de la fase móvil A, se agita la mezcla en un vórtex durante 5 s, y se inyectan 10 μL en el cromatógrafo.

### Estudio de validación

Los procedimientos de medida desarrollados basados en la UHPLC-MS/MS se han validado siguiendo las guías de la Agencia Europea del Medicamento (EMA) [18] y del Instituto de Normas Clínicas y de Laboratorio (CLSI) [19].

### Selectividad

Para el estudio de la selectividad se procesan muestras de blanco dobles (que no contienen ni ETP ni su PI); muestras de blanco (que presentan únicamente el PI); muestras cercanas al límite inferior de cuantificación (LLOQ) y 18 muestras diferentes de plasma y de líquido ascítico de pacientes no tratados con ETP que llegan al laboratorio de urgencias. De las muestras de plasma, seis presentan una visible turbidez (índices de lipemia con valores incluidos entre 114 y 395), seis están hemolizadas (índices de hemólisis comprendidos entre 27 y 165) y, para las otras seis, no se observa una interferencia aparente (índices de hemólisis y de lipemia inferiores a 6 y 12, respectivamente). Para las muestras de líquido ascítico, cuatro presentan una visible hemólisis (índice de hemólisis comprendidos entre 20 y 150), dos una visible turbidez (índices de lipemia de 103 y 202) y diez no muestran una interferencia visible (índices de hemólisis y de lipemia inferiores a 8 y 15, respectivamente).

Los índices de hemólisis y de lipemia se obtienen procesando las muestras por el analizador Cobas^®^ 6000 de Roche Diagnostics (Risch-Rotkreuz, Suiza).

Se acepta la ausencia de componentes interferentes si el área bajo la curva (AUC) del pico de todos los posibles picos interferentes, y para cada una de las muestras procesadas en el tiempo de retención del ETP, es inferior al 20 % de la AUC a las muestras cercanas al LLOQ para el ETP y del 5 % para el PI (ETP-D_4_) [18].

### Especificidad

Para el estudio de la especificidad se lleva a cabo un estudio similar al de la selectividad, pero variando el tipo de muestras de pacientes a utilizar. En este caso, se utilizan 10 muestras diferentes de plasma y líquido ascítico de pacientes polimedicados que recibían tratamiento antibiótico con ampicilina, aztreonam, cloxacilina, cefepima, ceftazidima, ceftriaxona, daptomicina, dalbavancina, meropenem y piperacilina.

La ausencia de componentes interferentes se da por válida si las AUC de todos los posibles picos interferentes para cada una de las muestras en el tiempo de retención del ETP es inferior al 20 % de las AUC correspondientes al LLOQ para el ETP, y del 5 % para su PI [18].

### Efecto matriz y recuperación de las muestras pretratadas

Para conocer el efecto de matriz (ME) y la recuperación (RE) tanto en plasma como en líquido ascítico se utilizan tres grupos de muestras diferentes a valores de 1,50, 10,0, 50,0 y 75 mg/L para el ETP y an un valor de 30 mg/L, para el PI. Estos tres grupos de muestras son: soluciones primarias de ETP (y su PI) diluidas con la fase móvil A (Muestras A); seis muestras de plasma (y líquido ascítico) de pacientes diferentes que no contienen ETP y a las que se les añade este antibiótico después del proceso de precipitación de proteínas (Muestras B); y las mismas seis muestras a las que se les adiciona ETP antes de la precipitación de proteínas (Muestras C). Todos estos grupos de muestras se procesan aleatoriamente y por quintuplicado.

Se calcula el ME y la RE (en %) como:

$$\text{ME }\left(\%\right)=\frac{\text{Media}\;{\text{AUC}}_{\text{Muestras}B}}{\text{Media}\;{\text{AUC}}_{\text{Muestras}A}}∙100$$

$$\text{RE }\left(\%\right)=\frac{\text{Media}\;{\text{AUC}}_{\text{Muestras}C}}{\text{Media}\;{\text{AUC}}_{\text{Muestras}B}}∙100$$

También se calculan los ME y RE normalizados dividiendo los valores de ME y RE obtenidos para el ETP entre los del PI.

El sesgo relativo obtenido debe incluirse en el intervalo ±15 % respecto a la concentración nominal, y la imprecisión no debe ser superior al 15 % [18]. Por otro lado, la variación de los ME y RE obtenidos para todas las concentraciones evaluadas debe ser inferior al 15 % [19].

### Curva de calibración

La integración de las AUC de los picos suavizados, las curvas de calibración y el cálculo de las concentraciones de ETP se establecen utilizando el software MassLynx™ v4.1 (Waters).

Las curvas de calibración se generan mediante un ajuste lineal entre el cociente AUC-ETP/AUC-ETP-D_4_ multiplicado por la concentración de ETP-D_4_ (eje y), frente a la concentración nominal de ETP (eje x). Las regresiones lineales se estiman mediante un modelo ponderado de ajuste 1/X, excluyendo la opción de forzar la curva de calibración por la ordenada en el origen.

Todas las concentraciones calculadas de los materiales de calibración deben estar comprendidos en el intervalo de ±15 % respecto a sus correspondientes concentraciones nominales (±20 % en el caso de la concentración cercana al LLOQ) [18].

### Precisión y veracidad

Para estimar la precisión intra- e inter-diaria (como el coeficiente de variación, CV) y la veracidad (como el sesgo relativo, *δ*_r_) se utilizan todos los QC preparados. Se procesan 30 alícuotas de cada QC repetidamente en un misma serie y día; y durante 60 días no consecutivos a lo largo de un mes y medio, respectivamente.

Para el cálculo del CV y *δ*_r_ a valores cercanos al LLOQ se realiza el mismo procedimiento descrito anteriormente, pero procesando materiales preparados a concentraciones similares a 0,50 mg/L.

Los CV obtenidos deben ser ≤15 % y los *δ*_r_ de ± 15 %. Además, en el caso del LLOQ, la relación señal/ruido (S/N) debe ser ≥5 y presentar un CV≤20 % y un *δ*_r_ de ± 20 % [18].

### Contaminación por arrastre

Para el estudio de la contaminación por arrastre se procesan muestras de blanco dobles (de plasma y líquido ascítico), muestras con valores cercanos al LLOQ y los materiales de calibración con mayor concentración en el siguiente orden:

*Muestra con valores de ETP cercanos al LLOQ − material de calibración con mayor concentración −* muestra de blanco doble.

La contaminación por arrastre es aceptable si el AUC del pico de ETP en las muestras de blanco doble son, como máximo, el 20 % del AUC del pico de ETP de la muestra en el LLOQ, y del 5 % del AUC del pico de PI [18].

### Integridad de la dilución

La integridad de la dilución se realiza procesando, seis veces, muestras de plasma y de líquido ascítico preparadas a valores dos veces el ULOQ y, posteriormente, diluidas diez veces con la muestra de blanco correspondiente. Seguidamente, se compara el valor medio obtenido con la concentración nominal.

El CV debe ser ≤15 % y *δ*_r_ de ± 15 % [18].

### Estabilidad

Para el estudio de la estabilidad de las soluciones primarias y secundarias, se realiza una preparación de estas, se diluyen apropiadamente con la fase móvil A. Posteriormente, estas soluciones se conservaron a (−75 ± 3) °C hasta su procesamiento tres meses después.

La estabilidad de las muestras pretratadas dentro del muestreador automático se comprueba reinyectándolas tras 6 h y 24 h posteriores a su almacenamiento a (15 ± 1) °C.

Los estudios de la estabilidad a corto y largo plazo se realizan utilizando los QC de ETP. Para la estabilidad a corto plazo, los QC se conservan a temperatura ambiente durante 2, 4 y 6 h, y posteriormente se procesan. Para la estabilidad a largo plazo, las muestras se congelan a (−75 ± 3) °C y se procesan tres mes después.

En todos los casos y utilizando diez réplicas, se estima la estabilidad en función del porcentaje de desviación (%D) del valor medio obtenido respecto a la concentración nominal:

$$\%D=\left(\frac{\text{Valor}\;\text{medio}\;\text{obtenido}\;\text{en}\;\text{las}\;10\;ré\text{plicas}-\text{Concentration}\;\text{nominal}}{\text{Concentration}\;\text{nominal}}\right)\cdot 100$$

El %D obtenido debe estar comprendido dentro del intervalo ±15 % [18].

### Aplicación clínica

Los procedimientos de medida basados en la UHPLC-MS/MS descritos se desarrollan y validan para dar soporte an un estudio de investigación sobre la eficacia del tratamiento de ETP en pacientes cirróticos con PBE. Este estudio ha sido aprobado por el Comité de Ética de nuestro hospital (JCA-ERT-2016-01) y cumple con los requisitos de la Asociación Médica Mundial y la Declaración de Helsinki.

### Pacientes

Son pacientes diagnosticados de cirrosis hepática con PBE nosocomial o relacionada con el sistema de salud ingresados en el Servicio de Aparato Digestivo de nuestro hospital.

### Administración de ertapenem

Los pacientes reciben una dosis de 1 g/24 h de ETP en infusión intravenosa durante 30 minutos. La duración del tratamiento está comprendida entre 5 y 7 días en función de la positividad de los hemocultivos y previa confirmación de curación citológica y microbiológica.

### Obtención de las muestras

Como el estado estacionario del ertapenem en el plasma se alcanza aproximadamente a las 20 horas del inicio del tratamiento [20], las muestras de pacientes se obtienen a las 48 h, 60 h, 72 h, 84 h, 96 h, 108 h y 120 h posteriores al inicio del tratamiento. Por el contrario, las muestras de líquido ascítico se recogen a las 48 h, 72 h, 96 h y 120 h después del inicio del tratamiento. Así, se obtienen las diferentes concentraciones de ertapenem “libres” en el estado estacionario (*f*ETPss). Para el cálculo de las *f*ETPss se asume un 95 % de unión a proteínas plasmáticas para el ETP [21].

Las muestras de plasma se han obtenido mediante venopunción y las muestras de líquido ascítico mediante paracentesis según práctica clínica habitual. Las condiciones de recogida y tratamiento de las muestras son las mismas que las descritas anteriormente.

## Resultados y discusión

### Desarrollo de los procedimientos de medida basados en la UHPLC-MS/MS

**Figura 1:**
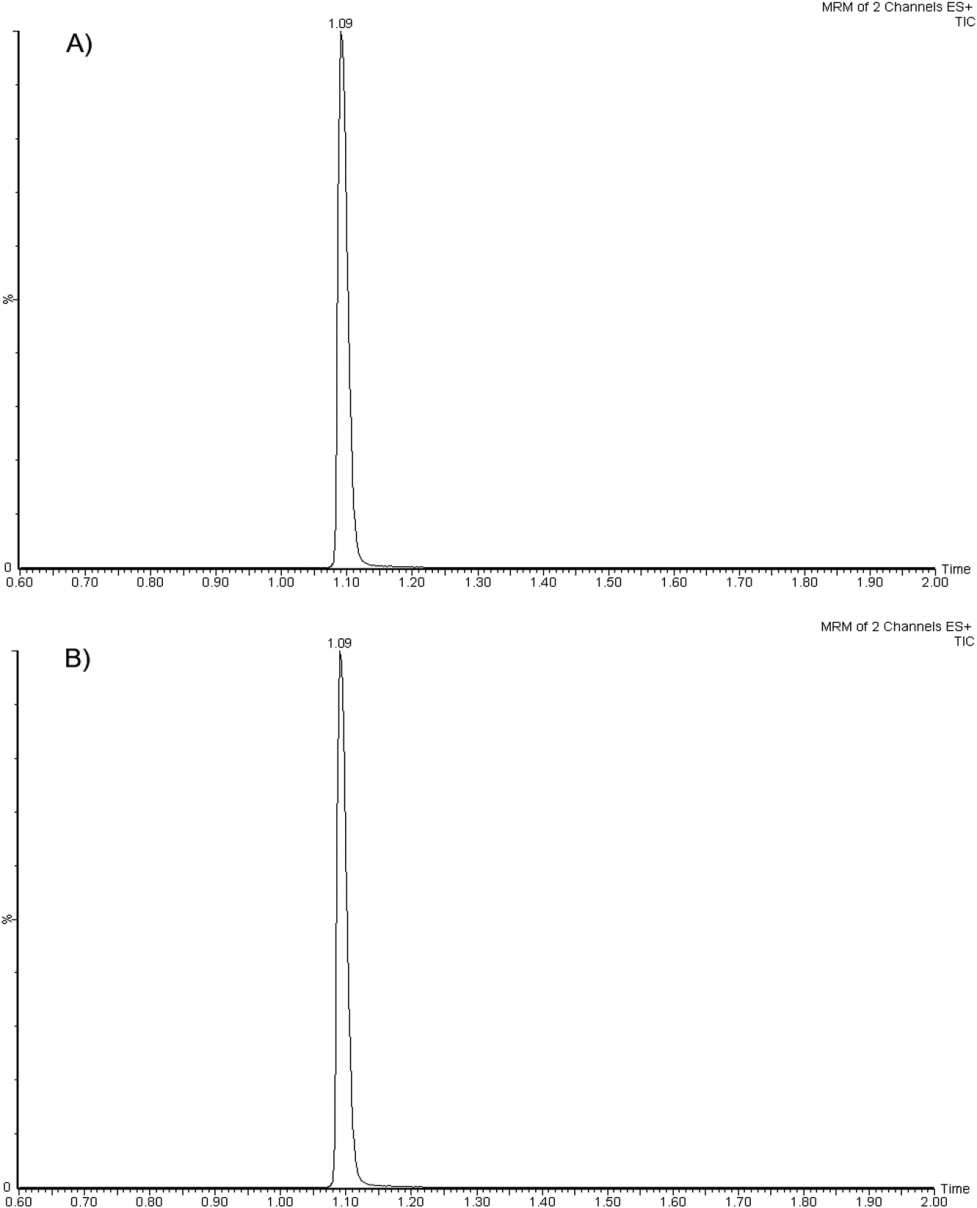
Cromatogramas representativos de ertapenem en (A) plasma y (B) líquido ascítico en un paciente cirrótico con peritonitis bacteriana espontánea que recibe una dosis intravenosa de 1 g/12 h de ertapenem durante 30 min.

Los tiempos de retención para el ETP y su PI son de 1,09 min con un tiempo total cromatográfico de 3,0 min, siendo ambos constantes y reproducibles (Figura 1). En la mayoría de los procedimientos publicados, el tiempo total cromatográfico varía entre los 2 y 15 min, que son similares o superiores al procedimiento de medida desarrollado [15], [16], [17], [18], [19], [20], [21], [22].

### Validación de los procedimientos de medida basados en la UHPLC-MS/MS

#### Selectividad y especificidad

En ninguna de las muestras procesadas se obtienen picos interferentes con AUC significativas en los tiempos de retención del ETP (valores comprendidos entre el 0,2 % y el 1,0 %) y de su PI (valores entre el 0,0 % y el 0,3 %).

### Efecto matriz y recuperación

En la Tabla 2 se muestran los resultados obtenidos para el ME y la RE. Los CV y *δ*_r_ obtenidos están comprendidos entre (4,3−11,0) % y (2,3−8,0) %, respectivamente.

**Tabla 2:** Efectos matriz, efectos matriz normalizados, recuperaciones y recuperaciones normalizadas para la concentración de ertapenem en el plasma y en el líquido ascítico.

Magnitud	Valor nominal, μg/L	Valor obtenido, μg/L	Efecto matriz (%)	Efecto matriz normalizado (%)	Recuperación (%)	Recuperación normalizado (%)
Pla – Ertapenem; c.masa	1,50	1,54	87,8 (11,9)	104,5 (12,6)	71,5 (11,0)	99,0 (12,4)
	10,0	10,5	85,3 (9,5)	101,5 (10,9)	73,2 (9,0)	101,4 (9,5)
	50,0	51,5	83,8 (7,9)	99,8 (8,4)	75,9 (8,0)	105,1 (8,8)
	75,0	76,7	81,2 (6,3)	96,7 (7,5)	76,4 (6,5)	105,8 (7,2)
LAs – Ertapenem; c.masa	1,50	1,56	92,5 (9,9)	100,8 (10,3)	70,3 (9,5)	98,8 (10,1)
	10,0	10,8	90,3 (7,6)	98,4 (8,4)	71,7 (8,2)	100,7 (8,8)
	50,0	53,6	87,4 (5,9)	95,2 (6,5)	72,6 (5,4)	102,0 (6,2)
	75,0	78,2	85,2 (4,5)	92,8 (5,8)	74,5 (4,3)	104,6 (5,0)

Entre paréntesis se indican los coeficientes de variación (en %) entre las diferentes muestras seleccionadas que han sido procesadas 5 veces cada una de ellas. Siguiendo las recomendaciones de la IUPAC y la IFCC [22]: LAs, líquido ascítico; Pla, plasma; c.masa, concentración de masa.

La utilización del PI seleccionado (ETP-D_4_) permite compensar la pérdida de ETP durante el proceso de pretratamiento de la muestra, con independencia de cuál sea el valor de la magnitud, y con una imprecisión aceptable (≤15 %). En cuanto a los valores de ME, se observa una supresión iónica para el ETP, que también es compensada por la utilización del PI.

### Curva de calibración

Todas las curvas de calibración obtenidas presentan un modelo de regresión lineal dentro del intervalo de medida establecido (0,50–100) mg/L. Los coeficientes de determinación obtenidos han sido superiores a 0,9945.

Los porcentajes de desviación obtenidos para las concentraciones calculadas de ETP en los materiales de calibración respecto a sus concentraciones nominales se encuentran entre el 5,1 % y el 13,1 % en el caso de los calibradores en plasma, y entre el 5,3 % y el 7,9 % para los calibradores en líquido ascítico.

### Precisión y veracidad

Los CV y *δ*_r_ están incluidos entre (3,9−17,3) % y (3,9−16,0) %, respectivamente (Tabla 3). Por otro lado, las relaciones *S/N* obtenidos para el LLOQ son≥10,5 para la concentración de ETP en el plasma, y≥12,2 en el caso de la concentración de ETP en el líquido ascítico.

**Tabla 3:** Valores de imprecisión y sesgo relativo intra- e interdiarios para los procedimientos de medida basados en la UHPLC-MS/MS que permiten medir la concentración de ertapenem en el plasma y en el líquido ascítico.

Magnitud Concentración nominal, g/L (tipo de muestra)	Intradiaria (n=30)			Interdiaria (n=30)		
	*x*‾, µg/L	CV, %	*δ*_r_, %	*x*‾ ± *s*, µg/L	CV, %	*δ*_r_, %
Pla – Ertapenem; c.masa						
0,50 (LLOQ)	0,55	14,2	10,0	0,58	17,3	16,0
1,50 (QC1)	1,61	10,9	7,3	1,64	14,5	9,3
10,0 (QC2)	10,5	7,2	5,0	10,9	10,7	9,0
50,0 (QC3)	52,5	5,5	5,0	54,2	7,8	8,4
75,0 (QC4)	77,2	4,7	2,9	80,2	5,9	6,9
LAs – Ertapenem; c.masa						
0,50 (LLOQ)	0,52	12,1	4,0	0,53	15,4	6,0
1,50 (QC1)	1,58	8,7	5,3	1,55	11,6	3,3
10,0 (QC2)	10,2	6,5	2,0	10,5	8,9	5,0
50,0 (QC3)	51,4	4,8	2,8	52,0	7,1	4,0
75,0 (QC4)	76,3	3,9	1,7	77,9	5,5	3,9

n, número de materiales procesados; *x‾,* media; CV, coeficiente de variación; *δ*_r_, sesgo relativo; LLOQ, límite inferior de cuantificación; QC, control interno de la calidad. Siguiendo las recomendaciones de la IUPAC y la IFCC [22]: LAs, líquido ascítico; Pla, plasma; c.masa, concentración de masa.

Los LLOQ, CV y *δ*_r_ obtenidos para la concentración de ETP en el plasma son similares o inferiores a los obtenidos por otros procedimientos de medida previamente descritos en la bibliografía [15], [16], [17], [18], [19], [20], [21], [22].

### Contaminación por arrastre

No se observan picos cromatográficos con AUC significativas en el mismo tiempo de retención del ETP y su PI. Para el ETP, la relación de las AUC de los picos cromatográficos obtenidas respecto a las AUC a valores cercanos al LLOQ son del 2,2 % para el plasma y 4,1 % para el líquido ascítico. Por otro lado, para el PI, estas relaciones de AUC son del 0,6 % y 1,0 %.

### Integridad de la dilución

Los CV resultantes en el estudio de la integridad de la dilución (dilución 1/10) son del 3,8 % en el caso de las muestras de plasma y del 2,8 % para las muestras de líquido ascítico. Por otro lado, los valores de *δ*_r_ obtenidos son de −1,8 % y −2,2 %, respectivamente.

### Estabilidad

Las soluciones primarias y secundarias son estables al menos 3 meses a (−75 ± 3) °C (%D ≤−3,9 % e ≤−4,3 %, respectivamente). En cuanto a los demás estudios de estabilidad realizados, las concentraciones de ETP son estables 6 h a temperatura ambiente (%D ≤−10,7 % para plasma e ≤−12,4 % para líquido ascítico), y 3 meses a (−75 ± 3) °C (%D ≤−6,9 % para plasma e ≤−8,8 % para líquido ascítico). También son estables en el muestreador automático durante 12 h a (15 ± 1) °C (%D ≤−13,8 % para plasma e ≤−14,5 % para líquido ascítico).

### Aplicación clínica

Las concentraciones de ERT en el plasma y líquido ascítico para los diferentes pacientes cirróticos con PBE se muestran en la Tabla 4.

**Tabla 4:** Características de los pacientes cirróticos con ascitis que presentan peritonitis bacteriana espontánea y sus concentraciones de ertapenem en el plasma y en el líquido ascítico obtenidas en diferentes periodos de tiempo.

Sujeto	Sexo	Edad, años	Peso, kg	FG, mL/min	Organismo aislado	CMI^a^, mg/L	Magnitud (resultados en mg/L)	48 h	60 h	72 h	84 h	96 h	108 h	120 h
1	Masculino	55	55	>90	*Staphylococcus aureus*	0,016	Pla – ETPss; c.masa Pla *– f*ETPss; c.masa	36,6 1,81	40,2 2,01	31,2 1,56	28,8 1,44	32,8 1,64	26,7 1,34	30,2 1,51
							LAs *–* ETPss; c.masa LAs *– f*ETPss; c.masa	18,7 0,935	– –	23,0 1,15	– –	23,4 1,17	– –	26,6 1,33
2	Masculino	62	82	>90	*Staphylococcus aureus*	0,030	Pla – ETPss; c.masa Pla – fETPss; c.masa	5,44 0,272	6,66 0,333	8,08 0,404	5,83 0,292	5,01 0,251	– –	– –
							LAs – ETPss; c.masa LAs – fETPss; c.masa	0,92 0,046	– –	1,33 0,067	– –	0,99 0,050	– –	– –
3	Masculino	55	101	70	*Enterobacter cloacae*	0,125	Pla – ETPss; c.masa Pla – fETPss; c.masa	33,4 1,65	34,2 1,71	33,2 1,66	34,5 1,73	37,3 1,87	35,4 1,77	– –
							LAs – ETPss; c.masa LAs – fETPss; c.masa	3,21 0,161	– –	3,54 0,177	– –	4,01 0,201	– –	– –
4	Femenino	65	65	82	*Klebsiella pneumoniae*	0,060	Pla – ETPss; c.masa Pla – fETPss; c.masa	4,52 0,226	5,24 0,262	7,28 0,364	8,05 0,403	12,2 0,610	18,1 0,905	– –
							LAs – ETPss; c.masa LAs – fETPss; c.masa	1,79 0,090	– –	2,22 0,111	– –	4,48 0,224	– –	– –
5	Femenino	54	54	>90	*Escherichia coli*	0,250	Pla – ETPss; c.masa Pla – fETPss; c.masa	9,11 0,455	8,22 0,411	8,38 0,419	7,34 0,367	7,54 0,377	8,83 0,442	9,21 0,461
							LAs – ETPss; c.masa LAs – fETPss; c.masa	7,14 0,357	– –	5,94 0,297	– –	5,64 0,282	– –	5,93 0,297

FG, filtrado glomerular estimado mediante la fórmula CKD-EPI; CMI, concentración mínima inhibitoria; ETPss, ertapenem en el estado estacionario; *f*ETPss, ertapenem no unido a proteína (“libre”) en el estado estacionario. ^a^Los valores de CMI para ertapenem se determinan mediante el método E-test^®^ (bioMérieux, Marcy-l’Étoile, Francia). Siguiendo las recomendaciones de la IUPAC y la IFCC [22]: LAs, líquido ascítico; Pla, plasma; c.masa, concentración de masa.

Los resultados muestran que el 100 % de las *f*ETPss alcanzan el objetivo comprendido entre el (50–100) % *f*T>CMI. La eficacia del tratamiento antibiótico, definida como curación citológica y microbiológica, se logra entre los días 5 y 6 para todos los pacientes evaluados.

## Conclusiones

Se desarrollan y validan procedimientos de medida basados en la UHPLC-MS/MS para la medición de la concentración de ETP en el plasma y en el líquido ascítico. Los resultados presentados muestran que los procedimientos son selectivos, específicos, con una adecuada capacidad de detección, exactos, veraces y precisos por lo que podrían ser utilizados para la realización de estudios PK/PD con fines de investigación. Además, éstos también podrían ser empleados para la monitorización farmacoterapéutica de ETP en la práctica clínica habitual.
